# Small-Scale Fisheries Bycatch Jeopardizes Endangered Pacific Loggerhead Turtles

**DOI:** 10.1371/journal.pone.0001041

**Published:** 2007-10-17

**Authors:** S. Hoyt Peckham, David Maldonado Diaz, Andreas Walli, Georgita Ruiz, Larry B. Crowder, Wallace J. Nichols

**Affiliations:** 1 Department of Ecology and Evolutionary Biology, University of California at Santa Cruz, Santa Cruz, California, United States of America; 2 Grupo Tortuguero A.C., La Paz, Baja California Sur, México; 3 Hopkins Marine Station, Stanford University, Pacific Grove, California, United States of America; 4 Grupo Tortuguero A.C., Distrito Federal, México; 5 Center for Marine Conservation, Nicholas School of the Environment and Earth Sciences, Duke University, Beaufort, North Carolina, United States of America; 6 California Academy of Sciences, San Francisco, California, United States of America; 7 Ocean Conservancy, Davenport, California, United States of America; Indiana University, United States of America

## Abstract

**Background:**

Although bycatch of industrial-scale fisheries can cause declines in migratory megafauna including seabirds, marine mammals, and sea turtles, the impacts of small-scale fisheries have been largely overlooked. Small-scale fisheries occur in coastal waters worldwide, employing over 99% of the world's 51 million fishers. New telemetry data reveal that migratory megafauna frequent coastal habitats well within the range of small-scale fisheries, potentially producing high bycatch. These fisheries occur primarily in developing nations, and their documentation and management are limited or non-existent, precluding evaluation of their impacts on non-target megafauna.

**Principal Findings/Methodology:**

30 North Pacific loggerhead turtles that we satellite-tracked from 1996–2005 ranged oceanwide, but juveniles spent 70% of their time at a high use area coincident with small-scale fisheries in Baja California Sur, Mexico (BCS). We assessed loggerhead bycatch mortality in this area by partnering with local fishers to 1) observe two small-scale fleets that operated closest to the high use area and 2) through shoreline surveys for discarded carcasses. Minimum annual bycatch mortality in just these two fleets at the high use area exceeded 1000 loggerheads year^−1^, rivaling that of oceanwide industrial-scale fisheries, and threatening the persistence of this critically endangered population. As a result of fisher participation in this study and a bycatch awareness campaign, a consortium of local fishers and other citizens are working to eliminate their bycatch and to establish a national loggerhead refuge.

**Conclusions/Significance:**

Because of the overlap of ubiquitous small-scale fisheries with newly documented high-use areas in coastal waters worldwide, our case study suggests that small-scale fisheries may be among the greatest current threats to non-target megafauna. Future research is urgently needed to quantify small-scale fisheries bycatch worldwide. Localizing coastal high use areas and mitigating bycatch in partnership with small-scale fishers may provide a crucial solution toward ensuring the persistence of vulnerable megafauna.

## Introduction

Though the unintended catch *(bycatch)* of industrial-scale fisheries can cause declines in migratory megafauna including seabirds, marine mammals, and sea turtles [1]–[7], the bycatch of small-scale fisheries has been overlooked. Small-scale fisheries, including artisanal, traditional and subsistence fisheries, occur in coastal waters worldwide, employing over 99% of the world's 51 million fishers [8]. But bycatch assessment and mitigation has focused on industrial rather than small-scale fisheries because the magnitude of industrial operations can yield high total bycatch, and data have not been available for small-scale fisheries [9].

Small-scale fisheries occur primarily in developing nations, and their documentation and management are limited or non-existent [10], [11], precluding evaluation of their impacts on non-target megafauna in coastal waters. New telemetry data reveal that migratory megafauna frequent coastal high use areas well within the range of small-scale fisheries, potentially producing high bycatch mortality with grave conservation consequences for vulnerable populations [12], [13].

Because many migratory megafauna are declining yet have ecological, economic, and cultural importance [5], [14], [15], assessing and mitigating bycatch that threatens them is a global conservation priority [4], [6]. Many species of migratory megafauna have delayed reproduction and low fecundity, making their populations vulnerable to bycatch of reproductively-valuable, late juvenile and adult stages [16], especially where they overlap with intense fisheries.

As a case study, we quantified the impacts of small-scale fisheries bycatch on the North Pacific loggerhead turtle population. North Pacific loggerheads nest exclusively in Japan, and annual censuses indicate as much as a 90% decrease in nesting females within the past three generations to fewer than 1000 yr^−1^, qualifying the population for critically endangered status [17]. Their juvenile stage lasts several decades during which turtles can migrate across the North Pacific [18], [19]. Extensive telemetry studies have recently revealed important foraging habitat for juvenile loggerheads in the central North Pacific [20], [21], and high levels of bycatch have been documented where industrial-scale fisheries overlap with this habitat both historically in drift gillnets [22] and more recently in longline fisheries [7].

Although the impact of small-scale fisheries on this population has not been quantified, reports indicated that juvenile loggerheads aggregate off of Baja California Sur, Mexico (BCS) exposing them to mortality in coastal fisheries operating from small (6–8 m) skiffs up to 55 km offshore [18], [23]. Small-scale fishing generates important income in BCS, but due to overfishing, landings and profits are dwindling [24]. Local fishers reported unintentionally catching dozens of loggerheads day^−1^ skiff^−1^, particularly while fishing bottom-set gear. Entangled and hooked turtles are generally drowned, and carcasses are discarded at sea [18]. We identified high use areas and quantified bycatch mortality of North Pacific loggerheads (*Caretta caretta*) in the small-scale fisheries of BCS and compared it with their bycatch in industrial-scale pelagic fisheries.

## Results

**Figure 1:**
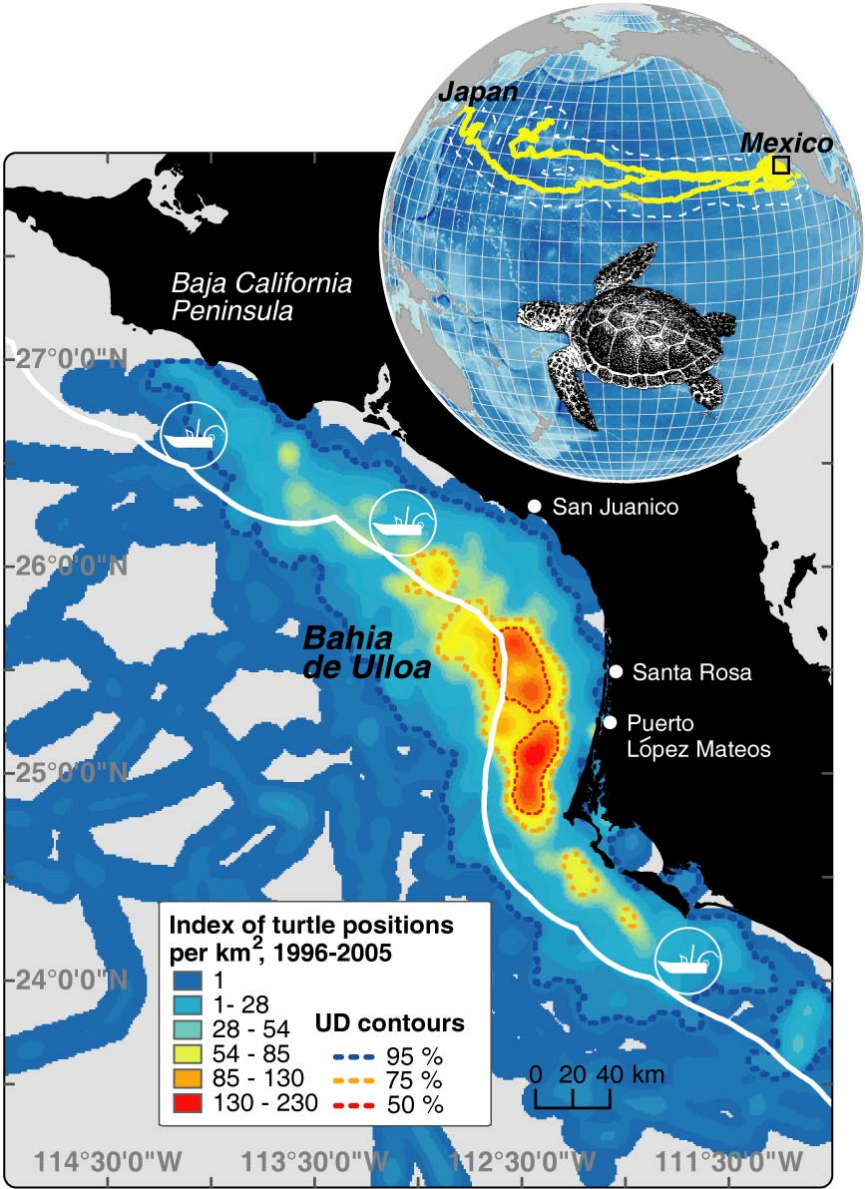
Kernel Density of Loggerhead Turtle Habitat Use in the North Pacific. *Inset:* Positions of tracked loggerheads (yellow) spanned the North Pacific Basin. The 50% utilization distribution for observed loggerheads consisted of an area of 4,115 km^2^ centered ∼32 km from the BCS coast, well within the 55 km range of small-scale fisheries (white line).

In partnership with local fishers, we used satellite telemetry to identify loggerhead high-use areas (or *hotspots)* and compared these with small-scale fishing grounds. We satellite-tracked 30 loggerhead turtles (curved carapace length (CCL); 72±9 cm, mean±SD; Table S1) from the Pacific coast of BCS from 1996–2005 to document loggerhead movement (mean track duration = 205±176 days and length = 5,041±4,460 km; Table S2). Though the observed range of tracked loggerheads spanned an area of ∼10^6^ km^2 ^across the North Pacific, turtles generally used a relatively small region during the 5,594 turtle days observed (Fig. 1). Only the four largest loggerheads (CCL = 88±7 cm) migrated from BCS waters towards Japanese nesting grounds; the other 26 turtles (CCL = 69±5 cm) spent 70.3%±25.8% (mean±SD between individuals) of their 4,059 observed days within the maximum range (55 km) of a dozen or more small-scale fishing fleets (Fig. 1 *inset*, Table S2).

We assessed loggerhead bycatch mortality by partnering with local fishers to observe two small-scale fleets that operated closest to the high use area. One fleet fished bottom-set gillnets (Puerto López Mateos) and the other bottom-set longlines (Santa Rosa; Fig. 1). In June-July 2005, we observed 11 loggerheads in 73 gillnet day-trips, or 0.16±0.7 loggerheads day^−1^. Eight of the 11 loggerheads were landed dead, resulting in an observed mortality rate of 73% in bottom-set gillnets. All loggerhead bycatch in bottom-set gillnets occurred during the 17 trips at the fleet's deepest of three fishing areas (32–45 m), where an average 0.65±1.3 loggerheads were caught per deep fishing trip.

In September 2005, we observed 26 loggerheads in seven longline day-trips (total 1,400 hooks) (Table S1). Loggerheads were caught on all observed longline trips (3.7±2.4 loggerheads day^−1^). Twenty-four loggerheads were landed dead or died shortly thereafter, resulting in an observed bycatch mortality rate of 92% during longline.

We estimated *minimum* annual loggerhead bycatch in each small-scale fishing fleet as the product of the observed mean of turtles killed per boat per day, the minimum number of boats fishing per day, and the minimum number of days fished per year. We estimated that in the 2005 season *at least* 299 and 680 loggerhead turtles died in the observed bottom-set gillnet and longline fleets, respectively. Our *minimum* estimate of total loggerhead mortality during 2005 in just two small-scale fishing fleets thus approached 1000 turtles. Although the estimates of *minimum* annual bycatch for the fleets we sampled are based on a limited number of fishing trips, actual loggerhead mortality for the region is likely to be much higher because 1) we used minimum values for all factors except bycatch rates, for which we used observed point estimates and 2) we estimated annual bycatch for two among twelve or more fleets which fish in or near the loggerhead high use area.

**Figure 2:**
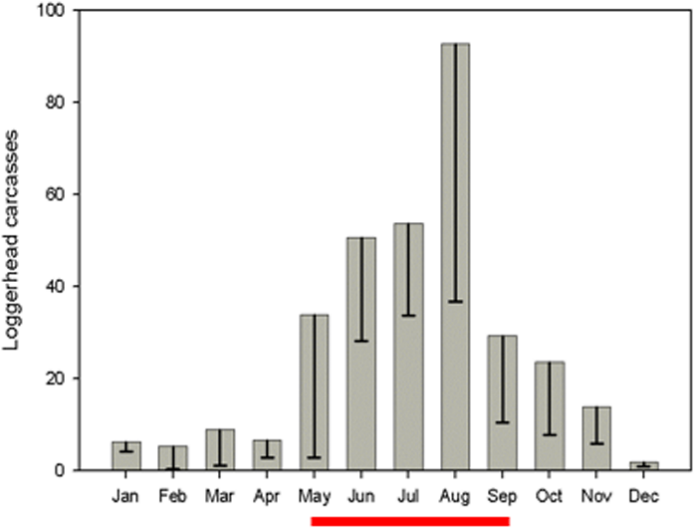
Loggerhead Carcasses Stranded at Playa San Lázaro 2003–5. 985 loggerhead carcasses stranded along the 43 km Playa San Lázaro from 2003–5. Nearly 80% (N = 781) of carcasses stranded from May-September, corresponding to seasonal operation of local small-scale fisheries (red line). Bars represent SD within months.

We also conducted daily (May-September) and weekly (October-April) shoreline surveys from 2003–2005 along the 43 km Playa San Lázaro, BCS, which is adjacent to the observed fishing grounds. Nearly 80% (N = 781) of the 982 loggerhead carcasses encountered were observed from May-September, corresponding to seasonal operation of local small-scale fisheries (Fig. 2). Carcasses were comprised of large juveniles or subadults (71±10 cm CCL; Table S1). In the Northwest Atlantic, only ∼15–30% of loggerhead carcasses discarded at sea strand, and the probability of stranding declines with distance from shore [25], [26]. Thus the 299 loggerhead carcasses that stranded during the months the fisheries operated in 2005 (May-September) likely represent a small fraction of discarded bycatch and corroborate our estimate of minimum bycatch mortality in 2005.

## Discussion

The long-term tracks of loggerhead turtles presented here plus the observed mortality confirm preliminary identification of a high use area for juvenile loggerhead turtles in the coastal waters of Baja California Sur, Mexico [18], [27]. The extended time periods over which juvenile loggerheads were tracked using this region suggests that it represents important developmental habitat for the population.

The US National Marine Fisheries Service noted that 37–92 large juvenile North Pacific loggerheads killed per year would “appreciably increase their extinction risk” [28]. Given that *minimum* annual loggerhead mortality due to bycatch in just two local BCS fleets is more than an order of magnitude greater, we conclude that these two fleets alone may threaten the persistence of the North Pacific loggerhead population.

Our minimum bycatch estimate (∼1000 loggerheads yr^−1^) for the two small-scale fleets rivals that of North Pacific industrial-scale fisheries. For example, the international pelagic driftnet fishery killed an estimated ∼800 loggerheads yr^−1^ until it was banned by international accord in 1992 [28]. The pelagic longline fishery was estimated to kill a minimum of 2600 loggerheads yr^−1^ across the entire Pacific Basin, roughly half of which (1300 loggerheads yr^−1^) may be killed in the North Pacific [7].

Bycatch per unit effort (BPUE) was at least an order of magnitude higher in the small-scale longline fleet (19.3 turtles per thousand hooks) than in Mexican and US pelagic longline fleets (0.00–1.40 loggerheads per thousand hooks) [9]. BPUE in the observed gillnet fishery (0.85 turtles gillnet km^−1^) was also more than an order of magnitude higher than that recorded for industrial-scale fisheries (0.01 turtles gillnet km^−1^) [22]. Furthermore, mortality of bycaught turtles was much higher in small- vs. industrial-scale fisheries (73–92% vs. 4–27%) [9], [22]. The disproportionately large impact of the two small-scale fisheries in this study is striking because of their spatially restricted, limited effort relative to the ocean basin-wide, massive effort of industrial-scale fisheries.

Because small-scale fisheries are conducted primarily in developing nations where management and enforcement are limited, assessing and mitigating their bycatch presents an international conservation challenge. Command-and-control approaches such as fisheries closures are often impractical and inadvisable, particularly in developing nations [8], [29], [30]. Because fishers' investment in the conservation process can increase their subsequent adoption of conservation strategies, solutions may depend on fishers' direct involvement and support in developing new social norms and economic alternatives [30], [31].

Accordingly, we forged innovative partnerships with local fishers and their families to assess and mitigate their bycatch [*32*]. From their participation in this research, fishers learned first-hand about the Pacific-wide impacts of their local bycatch and the potential for sustainable fishing and tourism in the newly identified hotspot. Concurrently we ran a bycatch awareness campaign using locally resonant media including murals, comic books, and regional festivals to celebrate loggerheads as a valuable resource and to empower fishers and their families as their stewards. As a result, fishers of Puerto López Mateos declared the core high use area a “Fishers' Turtle Reserve” in 2006. With the support of local, state, and federal governments, a coalition of fishers, managers, scientists, and citizens is now seeking federal legislation to establish and co-manage the reserve.

This case study demonstrates that a co-management strategy that directly engages local fishers and their families holds considerable promise in assessing and mitigating small-scale fisheries bycatch. Mexico is recognized worldwide for its successful protection of gray whales, and it has established numerous marine protected areas along the Baja California peninsula. The establishment of a co-managed loggerhead refuge would greatly reduce the extinction risk of this endangered population.

While bycatch in industrial-scale fisheries has driven declines in marine megafauna, small-scale fisheries can apparently have similarly severe effects where they overlap with megafauna high use areas. New telemetry studies are revealing that a range of migratory megafauna spend considerable time in coastal waters during vulnerable life history stages [12], [13], [33]. Furthermore, where quantified, small-scale fisheries are known to kill large numbers of non-target seabirds [34], marine mammals [35] and sea turtles [12] and to drive declines in megafauna target species [24].

Small-scale gillnet and longline fisheries are ubiquitous to coastal waters worldwide [8], [11] and can be expected to result in similarly high rates of bycatch mortality as exemplified by the two fleets observed in this study. Where small-scale fisheries and megafauna high use areas overlap worldwide, our case study showing population-level impacts of small-scale fisheries bycatch may be representative; small-scale fisheries may be among the greatest current threats to non-target megafauna. Further research is urgently needed to evaluate the impact of small-scale fisheries on vulnerable megafauna populations worldwide.

Although mitigating small-scale fisheries bycatch presents a daunting conservation challenge, the high BPUE of these fisheries provides an unexpected advantage. For each unit of small-scale fishing effort modified to reduce bycatch, a much higher benefit accrues for the megafauna than might be expected for industrial-scale fisheries. Localizing coastal distributional hotspots of vulnerable megafauna will be important for identifying previously unquantified bycatch mortality. Protecting coastal hotspots in partnership with local fishers may provide unforeseen leverage for ensuring the persistence of endangered marine megafauna.

## Methods

### Habitat use

The movements of loggerhead turtles were monitored using platform transmitting terminals (PTT) deployed on loggerhead turtles (n = 30) released along the Pacific coast of Baja California Sur (BCS), Mexico from 1996–2005. Twenty-seven of these turtles were captured by hand from small fishing boats and released within 18 hours and 10 km of capture. Two turtles were retrieved from bottom-set longlines on which they were shallowly hooked, instrumented, and released as above. One turtle was retrieved from gillnet fishers in the Gulf of California and held in captivity for 10 years before release.

We attached PTTs to turtle carapaces using polyester resin and fiberglass cloth [18] and monitored them via the Argos satellite system. We included all Argos-derived positions classified as 1, 2 or 3 in the spatial analysis. We filtered all other Argos positions (location classes A, B and O) based on a maximum rate of travel of 5 km h^−1^. Positions of location quality Z and those that clearly fell outside each turtle's track were omitted. In order to preserve the highest spatiotemporal resolution of the data, consecutive ARGOS hits were linearly interpolated to 3 positions per day based on great circle distances, based on the observed mean of 2.7±2.9 hits/day.

Multi-individual hotspots off the BCS coast were determined through an effort-weighted kernel density analysis of 9244 filtered positions to derive an index of turtle residence probability per unit area. From our dataset of filtered and interpolated positions, we derived an index of turtle residence probability per unit area as follows: 1) we extrapolated the number of turtle days spent per 0.01°×0.01° cell using kernel density analysis with a search radius of 0.5° and 2) we weighted the kernel density estimate of turtle days spent in each cell by multiplying it by the number of individual turtles using that cell. In this way we downweighted cells frequented by single or few individuals for extended periods to avoid biasing our identification of multi-individual high-use areas. We present turtle residence probability per cell as utilization distributions (UD) based on polygon coverage using least squares cross validation [36], [37] providing probability contours for the 50%, 75% and 95% UDs with the 100% contour reflecting the total range (Fig. 1).

### Fisheries observations

From June to July 2005, we made 73 day-long bottom-set gillnet trips with 5 fishing crews of Puerto López Mateos, the fishing community closest to the loggerhead high use areas identified in this study. Each boat fished a total of fifteen days across the fleets' range of gillnetting depths, with five days in each of three depth ranges that spanned the fleet's fishing grounds (5 to 18 m, 18 to 32 m, and 32 to 45 m) to standardize for boat-specific bycatch rates. Gillnet captains were compensated MN$500 per day, roughly 2/3 of daily gasoline expenditure for bycatch observations to be made.

The gillnet crews of Puerto López Mateos reportedly fish daily from May through August (70 to 110 day trips) in depths ranging from 5 to 45 m, with nets soaked for 20–48 hrs. Fishers worked from 6–8 m outboard-powered skiffs and targeted primarily California halibut (Paralichthys californicus, Ayres) and used 20.3 cm mesh monofilament gillnets of 400 m length and height from 3 m to 6 m. All nets were fished with “suspenders” connecting the float line to the sink line resulting in loose bags of net material. The fleet numbered up to 75 boats in 2005, with 9 to 40 boats fishing the fleet's deep area on a given day (32–45 m depths).

In September 2005, we made 7 daylong bottom-set longline trips with 5 local fishing crews from Santa Rosa. Longlines targeted shark species and were anchored in 60 to 90 m depths and checked each day. Crews checked and baited an average of 200 hooks per day.

The longline crews of Santa Rosa reportedly fished daily from August through September in 2005 (40 to 60 day trips), targeting primarily demersal sharks, with lines soaked 20–48 hours. Fishers used freshly caught mackerel or bycaught tuna or marlin for bait on “Japanese J-hooks” with inflected shanks. The Santa Rosa fleet numbered 5 to 6 boats in 2005.

In both fisheries we recorded the number, species, condition, and measurements of sea turtles captured.

### Shoreline mortality surveys

From January 2003 through December 2005, we conducted shoreline surveys on daily (May-September) and weekly (October-April) schedules along the 44 km Playa San Lázaro the shoreline closest to the loggerhead high use area described here. All turtle carcasses encountered were identified, measured, and marked. Data recorded on each stranded carcass included the following: observer name, stranding date, species, turtle number by day, location, curved carapace length and width (CCL and CCW), condition of carcass (decomposition state), tag numbers (if present), sex of carcass (when externally obvious), and observer notes. Curved carapace length was taken from the nuchal notch to the posterior marginal tip. Curved carapace width was taken at the widest part of the shell. All animals were painted and dragged well above the high tide line to avoid recounts.

### Estimating annual, local loggerhead bycatch

For the gillnet fishery, because bycatch was highly dependent on fishing depth, we multiplied the observed mean number of turtles caught per boat per day in depths from 32–45 m (0.65), by the reported minimum number of boats working waters deeper than 32 m on a given day (9; range 9 to 40) by the minimum number of days fished per year (70; range 70 to 110 trips) and discounted by the proportion of turtles released alive (27%). Based on this simple calculation we extrapolated a *minimum* annual bycatch mortality to be 299 loggerhead turtles for the 2005 season of the observed gillnet fleet*. For the bottom-set longline fishery, we multiplied the mean observed number of turtles caught per boat per day (3.7) by the minimum number of boats fishing (5; range 5 to 6) by the minimum number of days fished per boat in 2005 (40; range 40 to 55) and discounted by the proportion of turtles released alive (8%). We thus extrapolated a *minimum* annual bycatch mortality rate of 680 loggerhead turtles in the 2005 season for the observed bottom-set longline fleet*. Our *minimum* estimate of the total loggerhead mortality during 2005 for the two small-scale fishing fleets observed totaled ∼1000 loggerhead turtles.

### Comparing small- vs industrial-scale fisheries bycatch

The estimates of minimum annual bycatch and observed BPUE for the two fleets we sampled are based on relatively small numbers of fishing trips because of the inherent difficulties of documenting bycatch in small-scale fisheries [9], [18]. These difficulties include 1) logistical issues due to lack of space for observers on small-scale vessels, remoteness of fishing camps; 2) political issues due to lack of legal precedent for managing bycatch in small-scale fisheries and wariness of fishers; 3) sampling issues due to variability in gear, techniques, and effort both between and within fleets.

Gillnet bycatch estimates and BPUE for the Japanese pelagic driftnet fleet was published based on 25,500 km of observed gillnet sets [22], while we observed 58.4 km of gillnets (13.2 km of deep sets >32 m). Longline BPUE was reported for the US and Mexico fleets based on 1400×10^3 ^and 69×10^3^ hooks observed respectively [9], whereas we observed 1400 hooks.

Despite our relatively small sample sizes, we are confident that our samples are representative because 1) in semi-structured interviews we conducted in 2003, local fishers reported an average of 4 loggerheads caught per week per boat (roughly 0.65 per day-trip) and 2) in informal interviews made during this study longline fishers reported that the observed bycatch rates were normal. Furthermore, longline observations made both prior to and following this study showed similarly high bycatch rates.

### Participatory research

We partnered with fishers, community members, and managers to assess habitat use and bycatch and to design and conduct experiments to reduce turtle bycatch [38]. Complementing this research and drawing from the field of community-based social marketing [39]–[41], we designed a suite of outreach initiatives to empower fishers and their families to reduce bycatch [32]. Our approach grew from and was facilitated by the *Grupo Tortuguero,* a community conservation network that unites fishers and other conservationists of the Baja California peninsula and beyond [41]. Informative workshops for fishers and curriculum enrichment for schoolchildren conveyed the facts about bycatch. To supplement these experiences across whole communities, we offered a range of locally resonant media and formed local committees to organize public events such as regional festivals, parades, and sports competitions. Moreover, we partnered closely with local fishers and ecotour operators to demonstrate the feasibility of turtle and sportfishing tours as alternatives to depleted, high-bycatch fisheries.

## Supporting Information

Table S1 (0.03 MB DOC)

Table S2 (0.10 MB DOC)
