# Supplementary material for: Small-Scale Fisheries Bycatch Jeopardizes Endangered Pacific Loggerhead Turtles
Source: PLoS One. 2007 Oct 17;2(10):e1041. doi: 10.1371/journal.pone.0001041 (PMC2002513; doi:10.1371/journal.pone.0001041)
Supplement: Table S1 — (0.03 MB DOC) [file pone.0001041.s001.doc]

**Supplementary Table S1. Loggerhead Turtles Instrumented, Stranded, and Observed as Bycatch.**

| Source of turtles | N | mean  CCL (cm) | SD (cm) | range |
| --- | --- | --- | --- | --- |
| Captured and instrumented 1996-2005 | 30 | 73 | 9 | 38 |
| 4 largest of 30 instrumented | 4 | 88 | 7 | 17 |
| Strandings, Playa San Lázaro 2003-2005 | 982 | 71 | 10 | 61 |
| Bycatch, bottom-set gillnet | 11 | 78 | 8 | 21 |
| Bycatch, bottom-set longline | 26 | 79 | 6 | 22 |
