# Supplementary material for: Small-Scale Fisheries Bycatch Jeopardizes Endangered Pacific Loggerhead Turtles
Source: PLoS One. 2007 Oct 17;2(10):e1041. doi: 10.1371/journal.pone.0001041 (PMC2002513; doi:10.1371/journal.pone.0001041)
Supplement: Table S2 — (0.10 MB DOC) [file pone.0001041.s002.doc]

**Supplementary Table S2.** Loggerhead Turtles Instrumented with Platform Transmitting Terminals. Bold denotes the four turtles which migrated to or towards Japan.

|  | release |  |  | total | days within | % days within |
| --- | --- | --- | --- | --- | --- | --- |
|  | date | CCL | Tag | days | 55km of BCS | 55km of BCS |
| 1 | **10-Aug-96** | **86** | **7667** | **361** | **4** | **47.1** |
| 2 | 10-Aug-97 | 73 | 1085 | 268 | 80 | 29.7 |
| 3 | **26-May-98** | **98** | **3851** | **820** | **425** | **51.9** |
| 4 | 25-Jul-99 | 72 | 20750 | 352 | 39 | 11.1 |
| 5 | 25-Jul-99 | 64 | 20780 | 39 | 30 | 75.4 |
| 6 | 17-Aug-99 | 65 | 20779 | 282 | 227 | 80.6 |
| 7 | 27-Jul-00 | 64 | 21217 | 106 | 95 | 89.9 |
| 8 | 18-Jul-02 | 65 | 22180 | 96 | 83 | 86.1 |
| 9 | 3-Aug-02 | 60 | 22278 | 141 | 96 | 67.9 |
| 10 | 17-Aug-02 | 61 | 22279 | 142 | 12 | 8.7 |
| 11 | 26-Aug-02 | 72 | 22182 | 108 | 82 | 75.9 |
| 12 | 1-Sep-02 | 73 | 29068 | 171 | 136 | 79.6 |
| 13 | 2-Aug-03 | 63 | 17435 | 361 | 300 | 83.0 |
| 14 | 2-Aug-03 | 73 | 60249 | 137 | 87 | 63.6 |
| 15 | 9-Aug-03 | 79 | 21143 | 156 | 115 | 74.1 |
| 16 | 10-Aug-03 | 79 | 21129 | 227 | 223 | 98.1 |
| 17 | 13-Jul-04 | 66 | 42976 | 144 | 53 | 37.0 |
| 18 | 23-Jun-05 | 75 | 42977 | 206 | 114 | 55.6 |
| 19 | 23-Jun-05 | 76 | 42978 | 103 | 94 | 91.6 |
| 20 | 23-Jun-05 | 70 | 42979 | 192 | 144 | 75.0 |
| 21 | 24-Jun-05 | 75 | 42980 | 14 | 14 | 100.0 |
| 22 | 24-Jun-05 | 66 | 42981 | 115 | 94 | 81.7 |
| 23 | 30-Jun-05 | 73 | 42983 | 179 | 163 | 91.0 |
| 24 | 2-Jul-05 | 61 | 42985 | 4 | 4 | 100.0 |
| 25 | 2-Jul-05 | 71 | 42986 | 88 | 88 | 100.0 |
| 26 | 19-Aug-05 | 76 | 17710 | 148 | 102 | 68.8 |
| 27 | 19-Aug-05 | 75 | 60250 | 140 | 59 | 42.1 |
| 28 | 30-Aug-05 | 70 | 60247 | 140 | 88 | 62.9 |
| 29 | **2-Sep-05** | **88** | **16264** | **121** | **1** | **0.6** |
| 30 | **3-Sep-05** | **80** | **60248** | **135** | **33** | **24.4** |
